# The Impact of Interferon Beta-1b Therapy on Thyroid Function and Autoimmunity Among COVID-19 Survivors

**DOI:** 10.3389/fendo.2021.746602

**Published:** 2021-09-30

**Authors:** David Tak Wai Lui, Ivan Fan Ngai Hung, Chi Ho Lee, Alan Chun Hong Lee, Anthony Raymond Tam, Polly Pang, Tip Yin Ho, Chloe Yu Yan Cheung, Carol Ho Yi Fong, Chun Yiu Law, Kelvin Kai Wang To, Ching Wan Lam, Wing Sun Chow, Yu Cho Woo, Karen Siu Ling Lam, Kathryn Choon Beng Tan

**Affiliations:** ^1^ Department of Medicine, The University of Hong Kong, Queen Mary Hospital, Hong Kong, Hong Kong, SAR China; ^2^ Division of Chemical Pathology, Queen Mary Hospital, Hong Kong, Hong Kong, SAR China; ^3^ Department of Microbiology, The University of Hong Kong, Queen Mary Hospital, Hong Kong, Hong Kong, SAR China; ^4^ Department of Pathology, The University of Hong Kong, Hong Kong, Hong Kong, SAR China

**Keywords:** COVID-19, SARS-CoV-2, thyroid function tests, autoimmunity, interferon beta-1b, thyroid gland

## Abstract

**Background:**

Some studies have indicated that interferon (IFN) may be valuable in COVID-19. We aimed to evaluate the impact of short-term IFN on incident thyroid dysfunction and autoimmunity among COVID-19 survivors.

**Methods:**

We included consecutive adults without known thyroid disorder admitted to Queen Mary Hospital for COVID-19 from July 2020 to January 2021 who had thyroid function tests (TFTs) and anti-thyroid antibodies measured both on admission and at three months.

**Results:**

226 patients were included (median age 55.0 years; 49.6% men): 135 were IFN-treated. There tended to be more abnormal TFTs upon reassessment in IFN-treated patients (8.1% *vs* 2.2%, p=0.080). 179 patients (65.4% IFN-treated) had a complete reassessment of anti-thyroid antibodies. There were significant increases in titres of both anti-thyroid peroxidase antibodies (anti-TPO: baseline 29.21 units [IQR: 14.97 – 67.14] *vs* reassessment 34.30 units [IQR: 18.82 – 94.65], p<0.001) and anti-thyroglobulin antibodies (anti-Tg: baseline 8.23 units [IQR: 5.40 – 18.44] *vs* reassessment 9.14 units [IQR: 6.83 – 17.17], p=0.001) in the IFN-treated group but not IFN-naïve group. IFN treatment (standardised beta 0.245, p=0.001) was independently associated with changes in anti-TPO titre. Of the 143 patients negative for anti-TPO at baseline, 8 became anti-TPO positive upon reassessment (seven IFN-treated; one IFN-naïve). Incident anti-TPO positivity was more likely to be associated with abnormal TFTs upon reassessment (phi 0.188, p=0.025).

**Conclusion:**

IFN for COVID-19 was associated with modest increases in anti-thyroid antibody titres, and a trend of more incident anti-TPO positivity and abnormal TFTs during convalescence. Our findings suggest that clinicians monitor the thyroid function and anti-thyroid antibodies among IFN-treated COVID-19 survivors, and call for further follow-up studies regarding the clinical significance of these changes.

## Introduction

The coronavirus disease 2019 (COVID-19) pandemic, caused by severe acute respiratory syndrome coronavirus 2 (SARS-CoV-2), has infected more than 190 million people worldwide and caused more than 4 million deaths (1). Various therapeutic options are evaluated for COVID-19. Some studies have indicated that interferon may be valuable in COVID-19. Interferon beta-1b has been shown to shorten the duration of viral shedding, alleviate symptoms, reduce cytokine responses, and may reduce mortality and intensive care unit admission (2, 3).

Interferon beta-1b is an example of drug repurposing to fight against COVID-19 (4). Indeed, interferon beta-1b is a well-established therapy for multiple sclerosis (5). As a maintenance therapy used in a chronic setting, its long-term safety has been evaluated. Incident thyroid dysfunction and autoimmunity have been recognised in chronic interferon beta-1b therapy among patients with multiple sclerosis in retrospective and prospective, monocentric and multicentric studies (6). In contrast to the long-term treatment with interferon beta-1b in multiple sclerosis in terms of years, interferon beta-1b is given for a much shorter duration (typically a few days) in the context of acute COVID-19. Nonetheless, given the concerns of thyroid dysfunction and autoimmunity with interferon beta-1b in multiple sclerosis, it is prudent to investigate whether short-term interferon beta-1b therapy in acute COVID-19 is associated with thyroid dysfunction and autoimmunity. This will inform our clinical practice in the management of COVID-19 patients.

Hence, we carried out this prospective study of COVID-19 survivors to evaluate the impact of interferon beta-1b therapy on thyroid function and autoimmunity.

## Methods

The public health ordinance in Hong Kong required all patients tested positive for COVID-19 to be admitted to the hospital, including those detected on contact tracing and the Universal Community Testing Programme, regardless of symptoms (7). Our institution is one of the major centres in Hong Kong receiving confirmed COVID-19 patients. Consecutive adult patients (aged ≥18 years) admitted to our institution for COVID-19 between 21 July 2020 and 20 January 2021 were prospectively recruited. The presence of SARS-CoV-2 was confirmed in all patients by reverse transcription-polymerase chain reaction (RT-PCR) from the nasopharyngeal swab (NPS) or deep throat saliva (DTS), using the LightMix SarbecoV E-gene assay (TIB Molbiol, Berlin, Germany), which targeted the envelope protein (E) gene of SARS-CoV-2 (7, 8). Exclusion criteria were (i) history of thyroid, hypothalamic or pituitary disorders; (ii) use of anti-thyroid drugs or thyroid hormone replacement; and (iii) use of medications with potential impact on thyroid function, including systemic steroid, amiodarone, heparin and dopamine, before admission. Each patient had baseline blood tests taken within 24 hours after admission before starting COVID-19 treatments.

Serum thyroid-stimulating hormone (TSH), free thyroxine (fT4) and free triiodothyronine (fT3) were measured with immunoassays ADVIA Centaur^®^ TSH3-Ultra, FT4 and FT3 assays, respectively (Siemens Healthcare Diagnostics Inc., Erlangen, Germany). The reference ranges for TSH, fT4, and fT3 were 0.35–4.8 mIU/L, 12–23 pmol/L and 3.2–6.5 pmol/L, respectively. Anti-thyroglobulin (anti-Tg) and anti-thyroid peroxidase (anti-TPO) antibody titres were measured with QUANTA Lite^®^ Thyroid T and TPO enzyme-linked immunosorbent assay, respectively (Inova Diagnostics, San Diego, CA, USA). Positive anti-Tg and anti-TPO was defined by >100 World Health Organization (WHO) units (thereafter ‘units’), as specified by the manufacturer. Basic haematology and biochemistry panel, glycated haemoglobin (HbA1c) and inflammatory markers (C-reactive protein [CRP], erythrocyte sedimentation rate) were measured. Abnormal laboratory parameters were defined according to their respective reference ranges (7).

Demographics and significant comorbidities were recorded. Obesity was defined by the International Classification of Diseases, Ninth Revision, Clinical Modification (ICD-9-CM) code 278.0. Diabetes was defined by a known diagnosis of diabetes or HbA1c ≥6.5% on admission. Charlson comorbidity index was calculated for each patient. COVID-19-related symptoms were evaluated with a standard checklist. Respiratory rate, baseline oxygen saturation by pulse oximetry, and oxygen requirement on admission were captured. Cycle threshold (Ct) values were obtained from the qualitative LightMix SarbecoV E-gene assay (TIB Molbiol, Berlin, Germany) performed on specimens from NPS or DTS (whichever was lower) on admission. The Ct value represents the number of cycles required for a gene target or a PCR product to be detected. While viral loads were not directly measured with a dedicated quantitative RT-PCR assay in this analysis, studies have shown a good correlation between Ct values and SARS-CoV-2 viral loads (9, 10), such that the lower the Ct values, the higher the viral loads.COVID-19 severity was classified according to the ‘Chinese Clinical Guidance for COVID-19 Pneumonia Diagnosis and Treatment (7^th^ edition)’ published by the Chinese National Health Commission (11). Patients’ clinical outcomes were captured. For patients treated for COVID-19, one or more of the following were given: clofazimine (12), ribavirin, interferon beta-1b, or remdesivir (2). Dexamethasone (13) and subcutaneous low-molecular-weight heparin (LMWH) (14) were added at physicians’ discretion as clinically indicated. Interferon beta-1b was given once daily subcutaneously at a dose of 16 million IU. The decision to use interferon beta-1b was not influenced by the baseline thyroid function and antibody levels. The duration of interferon beta-1b therapy was recorded.

Follow-up visits were arranged around three months from admission to reassess thyroid function tests (TFTs) and anti-thyroid antibodies. Patients who had TFTs reassessed were included in the current study.

The study was approved by the Institutional Review Board of the University of Hong Kong/Hospital Authority Hong Kong West Cluster. Written consent has been obtained from each patient or subject after fully explaining the purpose and nature of all procedures used.

All statistical analyses were performed with IBM^®^ SPSS^®^ version 26. Two-sided p-values <0.05 were considered statistically significant. Data were presented as median with interquartile range (IQR) or number with percentage as appropriate. Between-group comparisons were performed with the t-test or Mann-Whitney U test for continuous variables as appropriate and Chi-square or Fisher’s exact tests for categorical variables as appropriate. Within-group comparisons were performed with paired t-test or Wilcoxon signed-rank test for continuous variables and McNemar’s test for categorical variables. Phi-coefficient was used to assess the relationship between two dichotomous categorical variables. Multivariable linear regression analysis was used to identify the independent determinants of changes in anti-TPO titre. All variables with statistical significance (p<0.05) in the univariate analysis were included in the multivariable regression analysis. Values not normally distributed were logarithmically transformed before analyses.

## Results

### Baseline Characteristics of the Cohort

A total of 226 patients were included, with their baseline characteristics summarised in **Table 1**. Their median age was 55.0 years, with no sex preponderance. Hypertension, diabetes and obesity were the most common comorbidities. The majority had non-severe acute COVID-19. 59.7% of the cohort received interferon beta-1b, and 16.4% received dexamethasone. The median duration of interferon beta-1b therapy was 5 days (range: 1 – 15 days; 65.9% received 5 days of interferon). Only 2.2% of the cohort required intensive care unit admission.

**Table 1:** Baseline characteristics of the cohort (n = 226).

	All	Interferon-treated	Interferon-naïve	P value
**Number**	226	135	91	—
**Baseline characteristics**				
Age (years)	55.0 (41.8 – 63.0)	56.0 (42.0 – 64.0)	54.0 (39.0 – 62.0)	0.297
Male	112 (49.6%)	66 (48.9%)	46 (50.5%)	0.807
Smoking	28/191 (14.7%)	18/112 (16.1%)	10/79 (12.7%)	0.511
Drinking	40/185 (21.6%)	21/106 (19.8%)	19/79 (24.1%)	0.488
Abnormal TFTs on admission	46 (20.4%)	31 (23.0%)	15 (16.5%)	0.235
Baseline anti-TPO positive	45 (19.9%)	26 (19.3%)	19 (20.9%)	0.765
Baseline anti-Tg positive	22 (9.7%)	16 (11.9%)	6 (6.6%)	0.191
COVID-19 severity				0.293
Mild	155 (68.6%)	97 (71.9%)	58 (63.7%)	
Moderate	61 (27.0%)	32 (23.7%)	29 (31.9%)	
Severe	10 (4.4%)	6 (4.4%)	4 (4.4%)	
SARS-CoV-2 PCR Ct value	24.10 (18.67 – 29.45)	21.77 (17.49 – 26.90)	28.10 (19.95 – 32.19)	**<0.001**
Lymphopenia	99 (43.8%)	67 (49.6%)	32 (35.2%)	**0.032**
CRP (mg/dL)	1.05 (0.31–2.83)	1.00 (0.31 – 2.82)	1.04 (0.31 – 3.06)	0.444
ESR (mm/hr)	40 (23 – 62)	39 (21 – 55)	46 (24 – 71)	0.068
**Comorbidities**				
Charlson comorbidity index				0.772
0	173 (76.5%)	104 (77.0%)	69 (75.8%)	
1	30 (13.3%)	18 (13.3%)	12 (13.2%)	
≥2	23 (10.2%)	13 (9.6%)	10 (11.0%)	
Hypertension	54 (23.9%)	33 (24.4%)	21 (23.1%)	0.813
Diabetes mellitus	32 (14.2%)	20 (14.8%)	12 (13.2%)	0.731
Obesity	17 (7.5%)	9 (6.7%)	8 (8.8%)	0.553
Malignancy	15 (6.6%)	7 (5.2%)	8 (8.8%)	0.286
CAD or heart failure	9 (4.0%)	5 (3.7%)	4 (4.4%)	0.999
Pulmonary disease	9 (4.0%)	5 (3.7%)	4 (4.4%)	0.999
Stroke or TIA	4 (1.8%)	3 (2.2%)	1 (1.1%)	0.650
**Clinical course**				
Length of hospitalisation (days)	8 (6–12)	9 (7 – 13)	6 (2 – 11)	**<0.001**
Oxygen requirement	27 (11.9%)	17 (12.6%)	10 (11.0%)	0.715
Intensive care unit admission	5 (2.2%)	4 (3.0%)	1 (1.1%)	0.651
Treatment				
Interferon beta-1b	135 (59.7%)	135 (100%)	0 (0%)	**<0.001**
Ribavirin	98 (43.4%)	98 (72.6%)	0 (0%)	**<0.001**
Remdesivir	54 (23.9%)	29 (21.5%)	25 (27.5%)	0.300
Dexamethasone	37 (16.4%)	25 (18.5%)	12 (13.2%)	0.288
Clofazimine	5 (2.2%)	4 (3.0%)	1 (1.1%)	0.651
SC LMWH	5 (2.2%)	4 (3.0%)	1 (1.1%)	0.651

Data presented as median with interquartile range or number with percentage as appropriate.

TFT, thyroid function test; anti-TPO, anti-thyroid peroxidase antibody; anti-Tg, anti-thyroglobulin antibody; COVID-19, coronavirus disease 2019; PCR Ct value, polymerase chain reaction cycle threshold value; CRP, C-reactive protein; ESR, erythrocyte sedimentation rate; CAD, coronary artery disease; TIA, transient ischaemic attack; SC LMWH, subcutaneous low molecular weight heparin, Values in bold represent statistical significance.

### Evolution of TFTs in the Cohort

**Figure 1:**
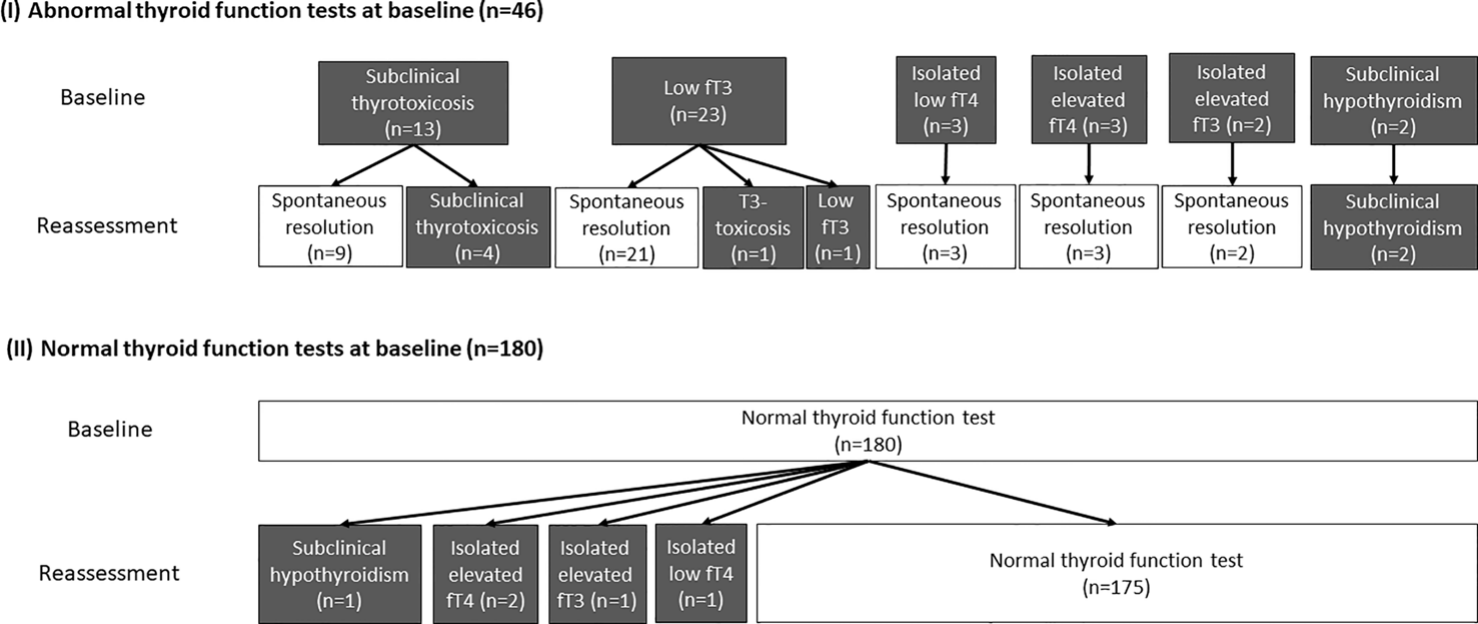
The evolution of the thyroid function of all 226 patients (grey boxes represent abnormal thyroid function while white boxes represent normal thyroid function).

Reassessment TFTs were performed at a median interval of 90 days (IQR: 68 – 105) after acute COVID-19. Out of the 46 patients who had abnormal TFTs upon admission, 38 (82.6%) recovered during convalescence. The evolution of thyroid function in the cohort is summarised in **Figure 1**. Four remained in subclinical thyrotoxicosis, defined by low TSH (i.e. <0.35 mIU/L) with normal fT4 and fT3 (i.e. fT4 and fT3 within their respective reference ranges). One had persistently low fT3 on reassessment, as he was admitted for fluid overload and clinically ill at the time of reassessment. One patient who initially had low fT3 developed T3-toxicosis (i.e. suppressed TSH <0.01 mIU/L with normal fT4 but elevated fT3 levels) at three months, followed by spontaneous resolution another three months later, suggestive of painless thyroiditis. Two patients had persistent subclinical hypothyroidism with positive anti-TPO, likely pre-existing Hashimoto’s thyroiditis diagnosed upon admission for COVID-19.

Among the 180 patients who had normal TFTs at baseline, 5 had abnormal TFTs upon reassessment: one patient had subclinical hypothyroidism; two patients had isolated mildly elevated fT4 levels; one had isolated mildly elevated fT3 levels; the remaining patient had isolated mildly low fT4 level.

Among all 226 patients, 135 were treated with interferon beta-1b. The comparison of the patients who were and were not treated with interferon is summarised in **Table 1**. Interferon-treated patients had higher SARS-CoV-2 viral load and more lymphopenia on admission. The length of stay of interferon-treated patients was longer. As ribavirin was given in combination with interferon, the interferon-treated patients were more likely to be treated with ribavirin. There tended to be more abnormal TFTs upon reassessment among interferon-treated patients (interferon-treated 11/135 [8.1%] *vs* interferon-naïve 2/91 [2.2%], p=0.080). Subgroup analysis of patients with normal TFTs at baseline (n=180) showed numerically more abnormal TFTs upon reassessment among interferon-exposed patients compared to those interferon-naïve (interferon-treated 4/104 [3.8%] *vs* interferon-naïve 1/76 [1.3%], p=0.308).

### Impact of Interferon beta-1b on Anti-Thyroid Antibodies

**Figure 2:**
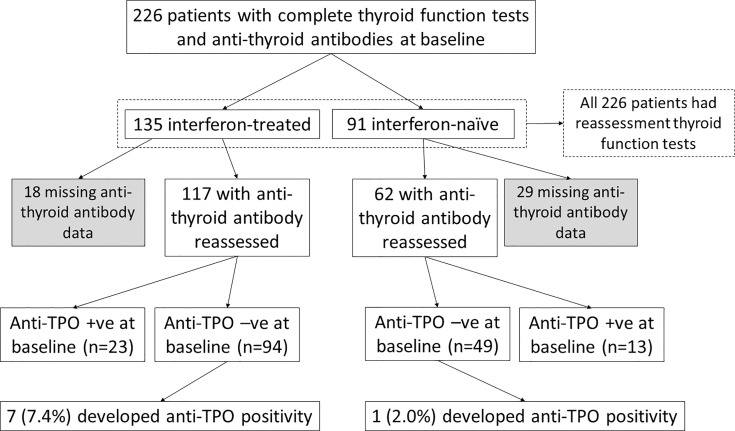
Flow diagram of the study.

**Figure 3:**
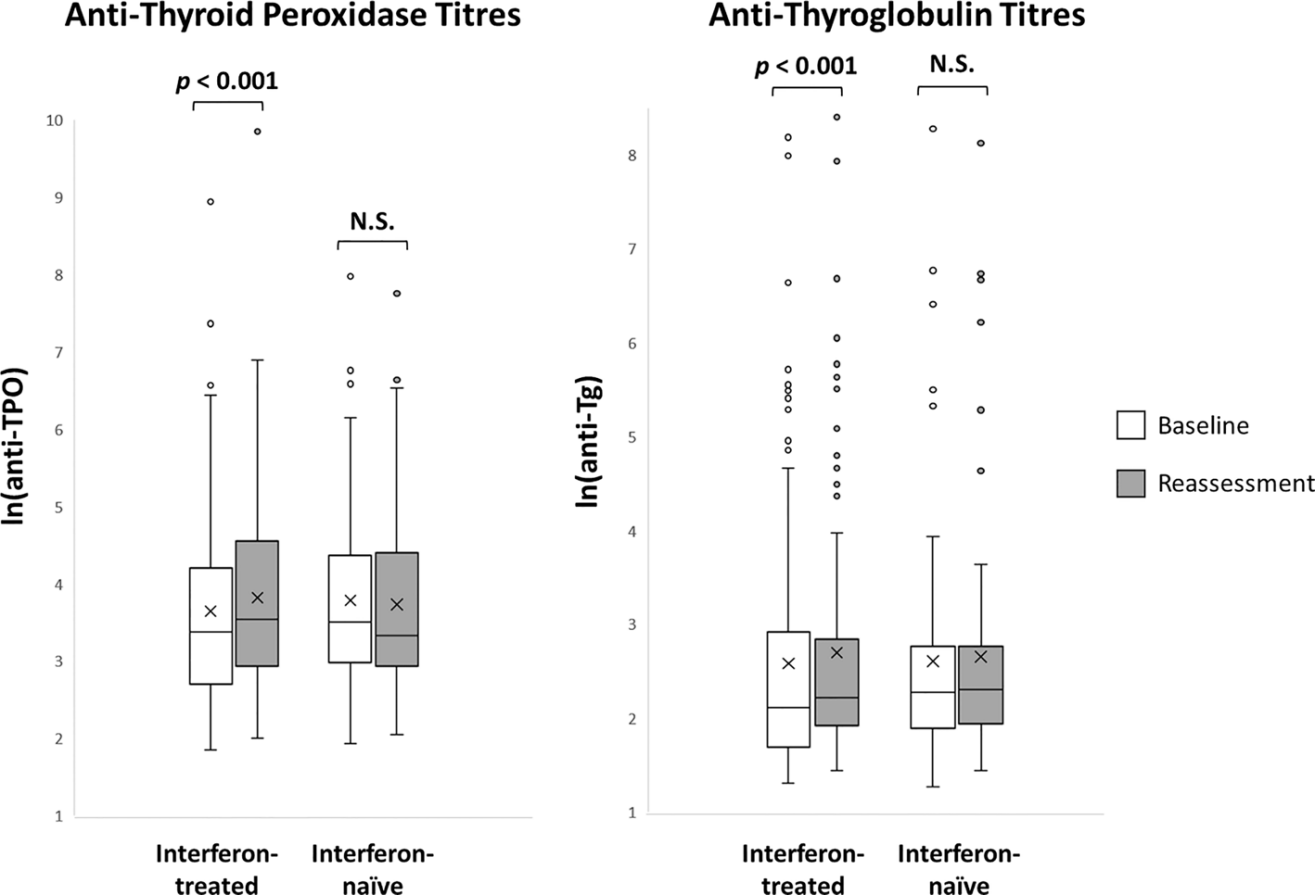
Anti-thyroid antibody titres at baseline and reassessment according to interferon exposure (n = 179). N.S., not significant.

The impact of interferon beta-1b on anti-thyroid antibodies was evaluated in 179 patients who had complete anti-TPO and anti-Tg results available at baseline and reassessment (**Figure 2**). One hundred seventeen patients were treated with interferon beta-1b, while 62 patients were interferon-naïve. There was no difference in age, sex, baseline COVID-19 severity, and baseline anti-TPO and anti-Tg positivity between interferon-treated and interferon-naïve patients. We first analysed the changes in anti-TPO and anti-Tg titres according to interferon exposure. (**Figure 3**) Among interferon-treated patients, both anti-TPO and anti-Tg titres showed statistically significant, but modest, increases upon reassessment. Anti-TPO titres increased from 29.21 units (IQR: 14.97 – 67.14) to 34.30 units (IQR: 18.82 – 94.65) (p<0.001). Anti-Tg titres increased from 8.23 units (IQR: 5.40 – 18.44) to 9.14 units (IQR: 6.83 – 17.17) (p<0.001). On the other hand, among interferon-naïve patients, titres of anti-TPO (baseline: 33.14 units [IQR: 19.77 – 79.12] *vs* reassessment: 27.80 units [IQR: 18.80 – 81.73], p=0.228) and anti-Tg (baseline: 9.76 units [IQR: 6.60 – 15.85] *vs* reassessment: 9.96 units [IQR: 6.92 – 15.89], p=0.908) did not significantly change upon reassessment. Changes in anti-TPO titres among the interferon-treated group were significantly different from the interferon-naïve group (interferon-treated: 3.75 units [IQR: -1.91 to 11.96] *vs* interferon-naïve: -2.63 units [IQR: -8.95 to 6.45], p=0.001). In contrast, the changes in anti-Tg titres were not significantly different between the two groups (p=0.294).

We further studied the factors associated with changes in anti-TPO titres upon reassessment. Among the continuous variables, age, baseline CRP or ESR, SARS-CoV-2 PCR Ct values, or length of hospitalisation did not correlate with changes in anti-TPO titres. Among the categorical variables, only interferon beta-1b treatment was significantly associated with changes in anti-TPO titres (p=0.001), but not sex, smoking/drinking, abnormal TFTs on admission, baseline COVID-19 severity, baseline anti-TPO positivity, baseline lymphopenia or comorbidities. Multivariable linear regression analysis showed that interferon beta-1b treatment (standardised beta 0.245, p=0.001) was a positive independent determinant of changes in anti-TPO titres.

To investigate the impact of interferon treatment on incident anti-TPO positivity, we analysed the 143 patients who were anti-TPO negative at baseline: 94 were interferon-treated, and 49 were interferon-naïve. Eight patients became anti-TPO positive upon reassessment (**Table 2**). There were more events of incident anti-TPO positivity in the interferon-treated group than in the interferon-naïve group, although not reaching statistical significance due to the small number of events (interferon-treated 7/94 [7.4%] *vs* interferon-naïve 1/49 [2.0%], p=0.264). Interestingly, with incident anti-TPO positivity, it was more likely to observe abnormal TFTs upon reassessment (phi 0.188, p=0.025).

**Table 2:** The thyroid function and antibody profile of patients who developed incident anti-TPO positivity upon reassessment.

Patient number	Sex/Age	On admission					COVID-19 treatment	Reassessment					
		TSH^a^	fT4^a^	fT3^a^	Anti-TPO^b^	Anti-Tg^b^		Days	TSH^a^	fT4^a^	fT3^a^	Anti-TPO^b^	Anti-Tg^b^
1	M/55	1.6	15	3.3	89.28	21.07	REM	71	3.7	14	3.7	**197.9**	38.01
2	M/58	0.55	16	**2.9**	64.92	**2957.1**	IFN+RIB+DEX	102	**0.02**	21	**7.2**	**100.12**	**2793.9**
3	F/67	1.3	18	N/A	45.45	4.31	IFN+RIB	89	1.7	17	5.3	**148.21**	6.58
4	F/61	0.61	19	4.1	44.36	5.10	IFN+RIB	109	0.45	18	4.7	**129.69**	5.62
5	F/61	1.2	17	3.5	6.85	4.90	IFN+RIB	93	2.8	20	5.0	**141.24**	8.31
6	M/21	1.1	17	4.4	99.85	6.62	IFN+RIB	105	0.47	19	5.2	**128.51**	9.67
7	M/51	2.2	16	4.6	97.06	4.84	IFN+RIB	90	1.7	20	5.6	**122.37**	7.31
8	M/45	1.6	13	4.9	82.88	8.97	IFN+REM+DEX	28	1.6	**11**	5.1	**108.09**	6.23

M, male; F, female; TSH, thyroid-stimulating hormone (mIU/L); fT4, free thyroxine (pmol/L); fT3, free triiodothyronine (pmol/L); anti-TPO, anti-thyroid peroxidase antibody (units); anti-Tg, anti-thyroglobulin antibody (units); COVID-19, coronavirus disease 2019; IFN, interferon beta-1b; RIB, ribavirin; REM, remdesivir; DEX, dexamethasone; N/A, not available.

Age expressed in years.

a Reference ranges: TSH 0.35 – 4.8 mIU/L, fT4 12–23 pmol/L, fT3 3.2–6.5 pmol/L.

b Positive anti-TPO defined by >100 units; positive anti-Tg defined by >100 units.

Values out of reference ranges are in bold.

## Discussion

We provided the first systematic analysis of the impact of COVID-19 treatment on the evolution of thyroid function and autoimmunity in patients with COVID-19. Our main finding was that interferon beta-1b treatment for COVID-19, even for such a short duration of a few days, could induce modest increases in anti-thyroid antibody titres and be associated with more incident anti-TPO positivity upon reassessment at three months. Furthermore, abnormal TFTs upon reassessment was more likely in interferon-treated patients and patients having incident anti-TPO positivity. Our findings would support the need for thyroid function and antibody monitoring in interferon-treated COVID-19 patients, and call for further follow-up studies regarding the clinical significance of these changes.

The interrelationship between thyroid and COVID-19 has become more evident with concerted efforts from research groups in basic science and clinical thyroidology (15, 16). With the expanding therapeutic armamentarium for COVID-19, the impact of COVID-19 treatment on the thyroid should be evaluated. Our current study focused on interferon beta-1b because of the concern about incident thyroid dysfunction and autoimmunity with its chronic use in multiple sclerosis. Some studies have indicated that interferon may be valuable in COVID-19, especially when initiated in the early stage of viraemia. Hung et al. have demonstrated early initiation of triple therapy (interferon beta-1b as the backbone) within one week from symptom onset to be effective (17). Similarly, a trial in Toronto evaluating peginterferon lambda treatment within one week from symptom onset has demonstrated efficacy in viral clearance (18). Although interferon use in the multinational SOLIDARITY trial has demonstrated no significant benefits in overall mortality, initiation of ventilation, and duration of hospital stay among hospitalised COVID-19 patients, the negative results could be explained by the probably late initiation of treatment (19). Antiviral agents are likely to be most effective during the early stage of viraemia, which is before the inflammatory pulmonary phase requiring hospitalisation (20). Hence, the treatments in the SOLIDARITY trial may not work in this late inflammatory pulmonary phase of the COVID-19. Furthermore, the patient characteristics in the SOLIDARITY trial were heterogeneous, and information on SARS-CoV-2 viral load was not available. As one of the potential treatment options of COVID-19, it is therefore clinically relevant to understand the impact of interferon treatment on the thyroid.

Earlier longitudinal studies of the impact of interferon beta-1b treatment among patients with multiple sclerosis reported up to 33% thyroid dysfunction and 20% thyroid autoimmunity, especially during the first year of treatment (21, 22). However, another study suggested only a random non-significant change in thyroid function, which did not correlate with thyroid autoimmunity (6). A subsequent larger cohort study of 106 patients with multiple sclerosis, with a much longer follow-up for up to 7 years, provided a clearer picture. Up to a quarter of patients developed incident thyroid dysfunction and autoimmunity, mainly during the first year of treatment with interferon beta (1a or 1b), supporting the need for thyroid function and antibody monitoring, especially during the first year (23). Among these, some developed permanent hypothyroidism. Moreover, pre-existing or incident thyroid autoimmunity was predictive of incident thyroid dysfunction (23). A further Italian long-term follow-up study evaluating various disease-modifying agents for multiple sclerosis confirmed the direct role of interferon therapy on the thyroid, showing a rate of around 10% for thyroid dysfunction and autoimmunity on treatment (24). Proposed mechanisms of the impact of interferon therapy on the thyroid include a direct inhibitory effect of interferon on iodine organification, especially in patients who develop hypothyroidism without antibody production (21); and the autoimmune reaction or immune system dysregulation associated with chronic interferon exposure (24). Compared to chronic use in multiple sclerosis, we have evaluated the impact of much shorter treatment duration (median of 5 days) and a more intense dosing regimen (16 million IU/day), in contrast to the usual regimen of 8 million IU every other day as a chronic therapy in multiple sclerosis. Intriguingly, even with such a short duration of interferon therapy, we still observed a 5.6% rate of incident anti-TPO positivity at three months, which correlated with a higher chance of abnormal TFTs during convalescence. Moreover, we noted a trend towards more abnormal TFTs upon reassessment in interferon-treated patients, although most of these were not clinically overt. Despite a modest magnitude of anti-TPO titre elevation among patients who developed incident anti-TPO positivity, further follow-up is warranted for potential subsequent thyroid dysfunction as the occurrence of anti-TPO can precede thyroid dysfunction (25). Hence, our findings would suggest the need for TFT and anti-thyroid antibody monitoring, especially in COVID-19 patients treated with interferon beta-1b. Further follow-up of these patients on a longer-term will elucidate whether this phenomenon is transient or permanent.

In our cohort, most interferon-treated patients were also treated with ribavirin, and none were treated with ribavirin alone. It could be difficult to tell whether interferon beta-1b alone or the interferon-ribavirin combination accounted for the increase in anti-thyroid antibody titres. Ribavirin is commonly used together with interferon in treating hepatitis C virus (HCV) infection. Ribavirin induces the production of T helper 1 (Th1) cytokines in the immune response against HCV. Combining ribavirin with interferon, therefore, stimulates the immune system response and eradicates HCV from the body. The Th1-like immune response involved has been shown to be a factor in the development and maintenance of organ-specific autoimmune diseases. Hence, ribavirin may be associated with the occurrence of autoimmunity (26). Nonetheless, a previous study has shown that adding ribavirin to interferon-alpha therapy in patients with HCV-related chronic hepatitis did not modify the anti-thyroid antibody pattern (27).

Studying the patterns of thyroid function and autoimmunity in the interferon-naïve group could facilitate our understanding of the potential of SARS-CoV-2 in triggering autoimmunity (28). In our cohort, we did not observe significant increases in anti-TPO and anti-Tg titres among the interferon-naïve group. There was only one patient having incident anti-TPO positivity among the 62 interferon-naïve patients. From our current prospective longitudinal observational study, there is no convincing evidence of COVID-19 triggering autoimmunity suggested by existing case reports of patients who developed Graves’ disease and Hashimoto thyroiditis following the diagnosis of COVID-19 (29). Nevertheless, SARS-CoV-2 infection may cause destructive thyroiditis (30), which may lead to subsequent development of autoimmunity, exemplified by cases of Graves’ disease (31) and Hashimoto thyroiditis (32) a few months after the initial episode of subacute thyroiditis (believed to be of viral origin). We have yet to observe these phenomena in our cohort, possibly related to the relatively short follow-up duration. Most patients with subclinical thyrotoxicosis in the acute COVID-19 in our cohort spontaneously normalised at three months. A longer-term follow-up of this cohort may shed light on this postulated sequela.

The strength of our study was the systematic reassessment of both thyroid function and anti-thyroid antibodies stratified by interferon exposure, showing an increase in anti-TPO and anti-Tg titres and incident anti-TPO positivity in the interferon-treated group, despite the anti-TPO and anti-Tg assays being semi-quantitative. The fact that the COVID-19 severity was non-severe for most patients in our cohort means that our results may be generalisable to COVID-19 patients at large. There are certain limitations in this study. Firstly, the sample size was relatively small, and there were missing data regarding anti-thyroid antibodies upon reassessment. Nevertheless, baseline characteristics including age, sex, COVID-19 severity, SARS-CoV-2 viral load and abnormal TFTs were comparable between those with and without anti-thyroid antibodies upon reassessment. Secondly, SARS-CoV-2 viral loads were represented by Ct values. Despite a good correlation (9, 10), direct quantitative measurements of viral loads would have been preferable if available. Thirdly, obesity was defined by the ICD-9-CM diagnostic code in our study as a categorical variable, instead of body mass index as a continuous variable, and was likely to be underreported. Fourthly, high-resolution computed tomography was done at the physicians’ discretion. Thus, the detection of imaging features of pneumonia in our cohort might be less sensitive. Fifthly, thyroid imaging was not available to assess the potential impact of interferon therapy or SARS-CoV-2 on the thyroid beyond TFT and anti-thyroid antibodies. Finally, the duration of follow-up was relatively short in the current study. The longer-term impact of interferon beta-1b will be informed with the continuation of follow-up of this cohort.

## Conclusion

Interferon beta-1b treatment, even when used in the short term for COVID-19, could induce modest increases in anti-thyroid antibody titres. It was also associated with incident anti-TPO positivity during convalescence, correlating with a higher likelihood of abnormal TFTs during convalescence. If short courses of interferon treatment become a standard therapy for COVID-19, the modest changes detected thus far may indicate a phenomenon of clinical importance. As for now, our findings suggest that clinicians monitor the thyroid function and anti-thyroid antibodies among interferon-treated COVID-19 survivors, and call for further follow-up studies regarding the clinical significance of these changes.

## Data Availability Statement

The raw data supporting the conclusions of this article will be made available by the authors, without undue reservation.

## Ethics Statement

The studies involving human participants were reviewed and approved by Institutional Review Board of the University of Hong Kong/Hospital Authority Hong Kong West Cluster. The patients/participants provided their written informed consent to participate in this study.

## Author Contributions

DL wrote the manuscript. DL, IH, CHL, AL, AT, PP, TH, CC, CYL, and WC researched the data. DL and CF performed statistical analyses. IH, CHL, AL, KKT, CWL, WC, YW, KL, and KCT critically reviewed and edited the manuscript. KCT initiated and supervised the study, is the guarantor of this work, has full access to all the data in the study and takes responsibility for the integrity of the data and the accuracy of the data analysis. All authors contributed to the article and approved the submitted version.

## Conflict of Interest

The authors declare that the research was conducted in the absence of any commercial or financial relationships that could be construed as a potential conflict of interest.

## Publisher’s Note

All claims expressed in this article are solely those of the authors and do not necessarily represent those of their affiliated organizations, or those of the publisher, the editors and the reviewers. Any product that may be evaluated in this article, or claim that may be made by its manufacturer, is not guaranteed or endorsed by the publisher.
